# Anatomical Variation in Root Traits Reflects the Continuum from Slow to Fast Growth Strategies Among Tropical Tree Species

**DOI:** 10.3390/plants14233590

**Published:** 2025-11-25

**Authors:** Jefferson Medina, Elizabeth Gusmán Montalván, Kerstin Pierick, Ángel Benítez, Nixon Cumbicus, Jürgen Homeier

**Affiliations:** 1 Maestría en Biología de la Conservación y Ecología Tropical, Universidad Técnica Particular de Loja, San Cayetano s/n, Loja 1101608, Ecuador; 2 Laboratorio de Ecología Tropical y Servicios Ecosistémicos—EcoSs_Lab, Departamento de Ciencias Biológicas y Agropecuarias, Universidad Técnica Particular de Loja, San Cayetano s/n, Loja 1101608, Ecuador; ecguzman@utpl.edu.ec; 3 Silviculture and Forest Ecology of the Temperate Zones/Spatial Structures and Digitization of Forests, University of Göttingen, Büsgenweg 1, 37077 Göttingen, Germany; kerstin.pierick@uni-goettingen.de; 4 Biodiversidad de Ecosistemas Tropicales-BIETROP, Herbario HUTPL, Departamento de Ciencias Biológicas y Agrpecuarias, Universidad Técnica Particular de Loja, San Cayetano s/n, Loja 1101608, Ecuador; arbenitez@utpl.edu.ec (Á.B.); nlcumbicus@utpl.edu.ec (N.C.); 5 Conservation Ecology, Philipps University of Marburg, Karl-von-Frisch-Straße 8, 35043 Marburg, Germany; jhomeie@gwdg.de; 6 Faculty of Resource Management, HAWK University of Applied Sciences and Arts, Daimlerstraße 2, 37075 Göttingen, Germany; 7 Plant Ecology and Ecosystems Research, University of Göttingen, Untere Karspüle 2, 37073 Göttingen, Germany

**Keywords:** anatomical traits, Ecuador, hydraulic strategies, interspecific variation, tropical tree species, resource acquisition

## Abstract

Root anatomical traits regulate water transport and resource acquisition in forest ecosystems, yet their variation and coordination with aboveground traits remain poorly understood in tropical forests. We investigated patterns of interspecific variation in four root anatomical traits (vessel diameter, vessel density, vessel lumen fraction, and theoretical hydraulic conductivity) across 20 tree species representing contrasting growth strategies in a premontane tropical forest of southern Ecuador. Using 160 root samples from transport roots (4–8 mm diameter), we quantified anatomical traits through microscopy and calculated theoretical hydraulic conductivity. We analyzed correlations with wood density and leaf functional traits and performed principal component analyses to assess trait coordination. Species exhibited substantial variation in root anatomical traits, ranging from acquisitive strategies with large vessel diameters (67.6 μm in *Ocotea* sp.) and high hydraulic conductivity (73.9 kg m^−1^ MPa^−1^ s^−1^ in *Alchornea glandulosa*) to conservative strategies with high vessel density (>185 vessels/mm^2^ in *Leonia crassa* and *Aspidosperma rigidum*). However, 60% of species displayed intermediate trait values, suggesting compensatory strategies rather than extreme specialization. We documented strong negative correlations between vessel diameter and both vessel density (r = −0.74) and wood density (r = −0.51), pointing at hydraulic efficiency-safety trade-offs. Principal component analysis revealed that leaf traits operated orthogonally to root anatomical traits, indicating independent axes of functional variation rather than coordinated whole-plant strategies. These decoupling challenges traditional plant economics spectrum assumptions and evidence that plants optimize above- and belowground functions through independent evolutionary pathways. Our findings highlight the prevalence of intermediate hydraulic strategies in tropical tree communities and provide new insights into the functional organization of diverse forest ecosystems.

## 1. Introduction

Plants possess adaptations that allow them to thrive in distinct environmental conditions. These adaptations are closely related to functional traits, including morphological, physiological, and phenological characteristics, which significantly influence the reproductive success and survival of species in their environment [1,2]. Root traits represent a key component of plant functional strategies [3,4,5,6], as they mediate belowground resource acquisition, determine water and nutrient uptake efficiency, and influence whole-plant carbon allocation patterns [6,7,8]. Despite their fundamental importance, information on how root anatomical traits vary among species and relate to hydraulic strategies remains limited [7,9,10], particularly in tropical forest systems where most research has focused on morphological rather than anatomical dimensions [11].

Anatomical traits of woody tissues, particularly vessel architecture, determine plant hydraulic performance and resource acquisition strategies [12]. Vessel diameter, vessel density, vessel lumen fraction, and hydraulic conductivity regulate water conduction efficiency in angiosperm species [12,13,14]. Among these, vessel diameter directly controls water transport capacity because it affects conduction efficiency through the fourth-power relationship described by the Hagen-Poiseuille equation [12], which in turn influences growth rates [13,14]. Wide vessels in stem wood provide higher conductivity per unit cross-sectional area compared to many small vessels [15], whereas a larger number of smaller vessels increases redundancy and reduces vulnerability to hydraulic failure [16]. More generally, water acquisition strategies of plants can, like nutrient acquisition strategies, be described in the framework of the plant economics spectrum, describing trade-offs between fast resource acquisition (acquisitive strategies) and tissue longevity (conservative strategies) [17]. These vessel-mediated trade-offs documented in stems extend to root systems [18,19,20], where anatomical traits serve as proxies for understanding resource acquisition processes and ecosystem-level functioning.

Root trait research has predominantly examined morphological dimensions in both temperate and tropical forests [21,22,23,24,25]. While fine roots are traditionally defined as those less than 2 mm in diameter [26], recent evidence demonstrates that functional criteria may be more appropriate for root classification than fixed diameter thresholds [27,28].

We adopt a functional approach targeting transport roots (4–8 mm diameter) to examine anatomical traits that coordinate with wood density in determining whole-plant hydraulic architecture [29]. However, root anatomical dimensions have received considerably less attention than morphological traits, with limited studies [30,31,32] often examining single traits in isolation or focusing predominantly on temperate species. This research gap is particularly pronounced for tropical systems, where quantifying interspecific variation in root anatomical traits is necessary to understand their ecological function and adaptive significance [7].

Premontane forests of southern Ecuador provide an optimal study ecosystem for investigating root trait variation because they harbor an exceptional species diversity across steep environmental gradients within relatively small spatial scales. These forests experience distinct climate and soil regimes that may drive divergent hydraulic strategies among coexisting species [33,34,35]. However, the systematic examination of root anatomical traits in relation to coordinated plant economic strategies in Neotropical forests is largely limited to morphological fine root traits [24,25,36].

In the present study, we investigated patterns of interspecific variation in four root anatomical traits related to hydraulic efficiency in 20 tree species with different life strategies in a premontane tropical forest in southern Ecuador. We addressed four specific research questions: (1) How do root anatomical traits (vessel diameter, vessel density, vessel lumen fraction, and hydraulic conductivity) vary among species with contrasting growth strategies? (2) To what extent do root anatomical traits correlate with wood density and leaf economic traits (specific leaf area and nitrogen content)? (3) How do species-specific combinations of root anatomical traits reflect coordinated hydraulic strategies? and (4) Do root anatomical traits determine species’ position along the fast-slow growth strategy continuum in tropical tree communities?

Based on established trade-offs in plant hydraulic architecture and resource economics theory, we predicted that anatomical features related to conduction would vary systematically among species according to their growth strategies. Specifically, we hypothesized that species with fast-growth strategies would exhibit wide vessels with higher hydraulic conductivity, combined with low wood specific gravity (WSG) and leaves with high specific leaf area (SLA) and nitrogen (N) values, to maximize resource acquisition and growth rates. Conversely, we expected that species with slow-growth strategies would display narrow vessels with lower hydraulic conductivity, higher WSG, and leaves with lower SLA and N values, reflecting resource conservation and stress tolerance mechanisms. This analysis of root anatomical traits and their coordination with aboveground traits across 20 tropical tree species provides the first comprehensive dataset on belowground anatomical strategies in Andean premontane forests, offering insights into plant trait coordination and species coexistence in these diverse tropical ecosystems.

## 2. Results

**Figure 1:**
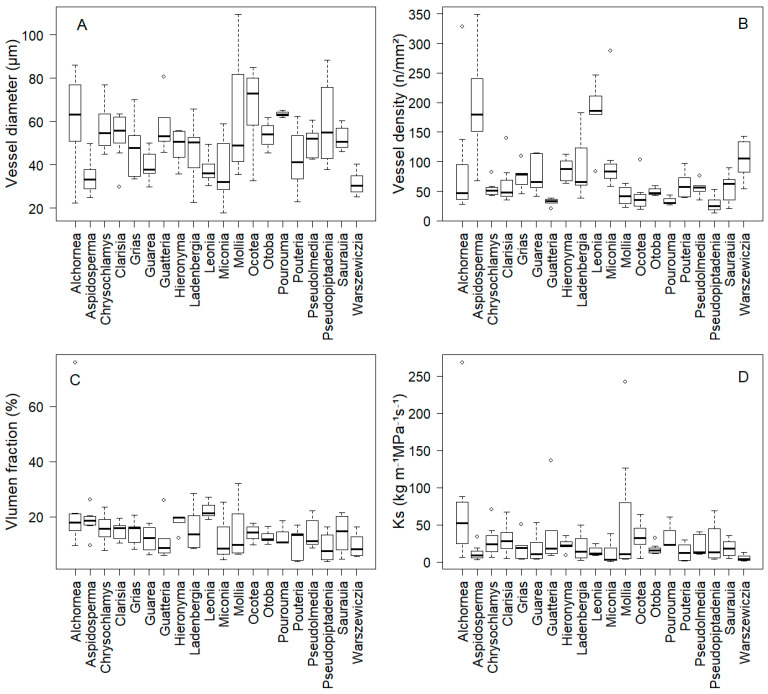
Interspecific variation in (**A**) vessel diameter (Vdia), (**B**) vessel density (Vdens), (**C**) vessel lumen fraction (Vlumen fraction) and (**D**) hydraulic conductivity (Ks) of 20 tree species. Box plots show median (central line), first and third quartiles (box boundaries), and 1.5× interquartile range.

Our results demonstrate substantial interspecific variation in root anatomical traits among the studied tree species. Concerning vessel diameter, *Ocotea* sp. exhibited the largest average at 67.6 μm, while *Warszewiczia coccinea* had the smallest at 31.5 μm (Figure 1A, Appendix A). However, the majority of species (70%) clustered within a relatively narrow range of 40–55 μm, defined as intermediate based on the interquartile range of our dataset (25th–75th percentiles; see Appendix A for species-specific values), suggesting that most species adopt intermediate vessel diameter strategies rather than extreme specialization (Figure 1A, Appendix A). These values fall within the range reported for tropical tree roots (25–80 μm) (Figure 1A).

In terms of vessel density, *Leonia crassa* and *Aspidosperma rigidum* showed the highest values, each exceeding 185 vessels/mm^2^, whereas *Pseudopiptadenia suaveolens* had the lowest density with an average of 27.8 vessels/mm^2^ (Figure 1B, Appendix A). Statistical analysis confirmed significant interspecific variation (F = 18.67, *p* < 0.001), with high-density species (>150 vessels/mm^2^) significantly differing from low-density species (<50 vessels/mm^2^). Regarding vessel lumen fraction, *Leonia crassa* presented the largest, occupying 22% of the root cross-sectional area, while *Pseudopiptadenia suaveolens* had the smallest at 8% (Figure 1C, Appendix A). Finally, hydraulic conductivity was highest in *Alchornea glandulosa* at 73.9 kg m^−1^ MPa^−1^ s^−1^, and lowest in *Miconia aff. punctata* at 11.1 kg m^−1^ MPa^−1^ s^−1^ (Figure 1D, Appendix A).

The species with the largest vessel diameters (*Ocotea* sp.) differed markedly from those with the highest vessel density (*Leonia crassa* and *Aspidosperma rigidum*), suggesting diverse strategies for water transport. The majority of species (65%) exhibited intermediate trait values, determined by values falling between the 25th and 75th percentiles across all measured traits (see Appendix A for species-specific values), suggesting that balanced hydraulic strategies predominate over extreme specialization in this forest community.

Additionally, the species showing extremes in lumen area (*Leonia crassa* and *Pseudopiptadenia suaveolens*) also exhibited contrasting patterns in vessel density, indicating clear trade-offs between these traits. The wide range in hydraulic conductivity across species further underscores the diversity of water uptake capacities in this forest ecosystem.

**Figure 2:**
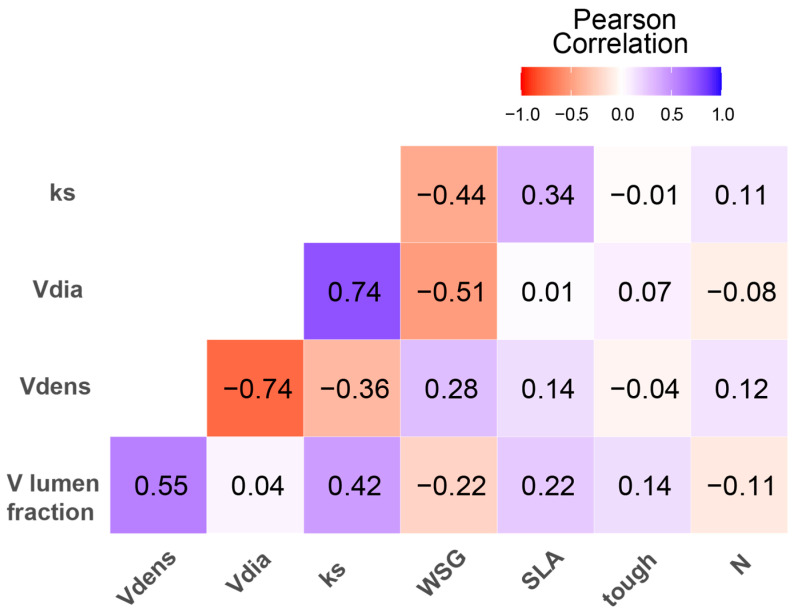
Correlation matrix showing Pearson correlation coefficients among root anatomical traits, stem wood density and leaf traits. Color intensity and square size represent correlation strength. Blue squares indicate positive correlations; red squares indicate negative correlations. Asterisks indicate significance levels.

The correlation matrix revealed several significant relationships among the studied variables (Figure 2).

Vessel diameter and hydraulic conductivity showed a strong positive correlation (*p* < 0.0001; r = 0.74), suggesting that larger vessel diameters are associated with higher hydraulic conductivity in the roots.

Furthermore, vessel diameter was negatively correlated with stem wood density (*p* < 0.002; r = −0.51), implying that species with higher wood density tend to have smaller root vessel diameters. Finally, vessel density was positively correlated with vessel lumen fraction (*p* < 0.01; r = 0.55), indicating that species with higher vessel densities also have larger lumen fractions in their root cross-sections.

Intraspecific variation was substantial across all measured traits, with coefficients of variation ranging from 15% to 35%. Vessel diameter showed the highest intraspecific variation (mean CV = 28%), followed by hydraulic conductivity (CV = 25%) and vessel density (CV = 18%). This considerable phenotypic plasticity suggests that individual trees may adjust their root anatomical traits in response to local micro-environmental conditions.

**Figure 3:**
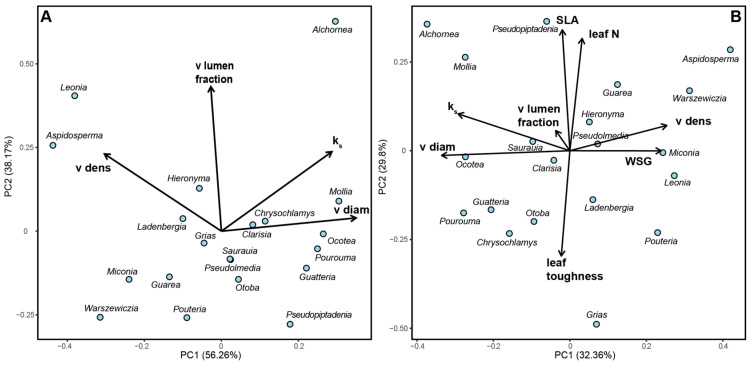
Principal component analyses showing the distribution of investigated tree species in trait space. (**A**) Based on root anatomical properties (PC1: 56.3% variance, PC2: 38.2% variance); PC1 represents hydraulic efficiency (positive loadings: vessel diameter +0.89, hydraulic conductivity +0.91), while PC2 represents hydraulic safety (positive loadings: vessel density +0.82, vessel lumen fraction +0.76). (**B**) Comparison of belowground and aboveground properties (PC1: 32.4% variance, PC2: 29.8% variance); PC1 is dominated by root anatomical traits and stem wood density, while PC2 is characterized by leaf properties. Species abbreviations follow Appendix B. Arrows indicate trait loadings, with length representing contribution strength. See Appendix C for complete loading scores.

The principal component analysis (PCA) revealed distinct patterns in the distribution of tree species based on their root anatomical traits and other characteristics. In the first PCA (Figure 3A), the first component explained 56% of the variance, with PC1 primarily representing hydraulic conductivity and vessel diameter. Species such as *Alchornea glandulosa*, *Mollia gracilis*, *Ocotea* sp., and *Pourouma cecropiifolia* were associated with high hydraulic conductivity and larger vessel diameters. In contrast, *Aspidosperma rigidum* and *Leonia crassa* were characterized by high vessel density.

The second PCA (Figure 3B) provided further insights into the relationships between various plant traits. The first axis, accounting for 32% of the explained variance, was primarily influenced by root anatomical traits and wood density. The second axis was characterized by leaf properties, vessel diameter, and hydraulic conductivity. This analysis demonstrates the coordinated variation between belowground and aboveground plant traits in shaping species ecological strategies.

Species such as *Alchornea* and *Mollia* showed a strong association with high hydraulic conductivity and large vessel diameters, positioning them on the positive end of PC1 in Figure 3A. Conversely, *Aspidosperma* and *Leonia* were positioned towards the negative end of PC1, indicating their association with high vessel density. In Figure 3B, the distribution of species along both axes reflects the varying influences of root anatomy, wood density, and leaf traits on species differentiation within this premontane tropical forest ecosystem.

These findings highlight the importance of considering multiple plant traits, both above- and belowground, in understanding species distribution and ecological strategies in tropical forest environments. The PCAs demonstrate that root anatomical traits play a crucial role alongside more traditionally studied characteristics like leaf properties and wood density in shaping plant community composition and function.

## 3. Discussion

The predominance of intermediate strategies over extreme specialization represents a fundamental finding of our study. The data presented in this research provide the first comprehensive report on anatomical root traits associated with native species of a premontane tropical forest in southern Ecuador. While we documented an extreme dichotomy between the most acquisitive and most conservative species, the majority of species (60%) adopted intermediate compensation strategies rather than extreme specialization. This pattern suggests that most tropical tree species optimize their hydraulic architecture through moderate trait combinations that balance efficiency and safety trade-offs [25,37].

The establishment of relative thresholds based on our dataset provides a framework for understanding the continuum of hydraulic strategies in tropical forests. Our statistical comparisons clearly demonstrate significant differences between the extreme ecological strategies. Species classified as acquisitive (*Alchornea glandulosa*, *Pourouma cecropiifolia*; see Figure 3A and Appendix A) exhibited the predicted combination of increased vessel diameter (>60 μm), low vessel density (<50 vessels/mm^2^) and high hydraulic conductivity (>35 kg m^−^^1^ MPa^−^^1^ s^−^^1^) in their roots, prioritizing rapid water uptake.

Generally, these species have high specific leaf areas, high foliar nutrient contents (nitrogen, phosphorus) and low leaf dry matter contents, a strategy that lowers leaf production costs and allows them to have high growth rates, dominating on fertile soils [7,25,36]. Conversely, species at the conservative extreme (*Aspidosperma rigidum* and *Leonia crassa*; Figure 1B and Figure 3A, Appendix A) display decreasing vessel diameter (<38 μm) high vessel density (>185 vessels/mm^2^) and low hydraulic conductivity (<15 kg m^−1^ MPa^−1^ s^−1^) in their roots, prioritizing resource conservation to tolerate stressful environmental conditions. Generally, these species are associated with low specific leaf area, high leaf dry matter content and low foliar nutrient concentrations, generating physically resistant leaf structures [7,25,36,38].

From a phylogenetic and evolutionary perspective within our study system, the distribution of hydraulic strategies across the 20 studied species representing 15 families suggests that vessel architecture has evolved independently multiple times in response to environmental filtering. The clustering of *Aspidosperma* (Apocynaceae) and *Leonia* (Violaceae) in the conservative strategy space, despite their distant phylogenetic relationships, indicates convergent evolution of similar hydraulic solutions. Similarly, the acquisitive species represent diverse families (Euphorbiaceae, Malvaceae, Lauraceae, Urticaceae), suggesting that wide-vessel, high-conductivity strategies have evolved as parallel adaptations to resource-rich environments [39]. However, broader phylogenetic analyses encompassing more diverse tropical lineages and biogeographic regions are necessary to confirm patterns of convergent evolution at larger taxonomic and spatial scales. Our findings should be interpreted as preliminary evidence for convergent evolution within this premontane forest system.

The orthogonality between leaf and root traits represents perhaps the most significant finding of our study. This decoupling challenges the traditional view of coordinated whole-plant economic strategies for all resources and suggests that plants may optimize water and nutrient economics strategies through independent evolutionary pathways [17,40]. This finding has profound implications for plant functional ecology, as it indicates that leaf economics spectrum predictions may not extend to root hydraulic architecture, and vice versa. The independence of these trait dimensions may reflect the different selective pressures operating above- and belowground: leaves respond primarily to light competition and carbon economy, while roots respond to water availability and soil heterogeneity [25,26].

This orthogonal relationship contrasts with findings from other forest ecosystems. In temperate forests, stronger coordination between leaf and root economics has been reported, with root branching patterns representing a leading trait of the plant economics spectrum and species showing more integrated whole-plant strategies [41,42]. Studies in subtropical forests have also documented positive correlations between above- and belowground resource acquisition strategies, suggesting coordinated whole-plant economics [41]. The decoupling observed in our premontane tropical forest system may reflect ecosystem-specific adaptations to the unique conditions of montane tropical environments, where high species diversity, steep environmental gradients, and year-round water availability create selective pressures that favor functional flexibility over coordinated specialization. This suggests that the degree of trait coordination may vary systematically across forest biomes, with diverse tropical montane systems exhibiting greater independence between above- and below-ground trait dimensions.

While our study focused on interspecific variation, it is important to acknowledge that intraspecific variation in root anatomical traits may also be substantial. The coefficient of variation within species ranged from 15 to 35% for most traits, suggesting considerable plasticity within species. This variation could be attributed to micro-environmental differences within our study plots, individual tree age, or phenotypic plasticity in response to local soil conditions.

Our results confirm the fundamental trade-off between hydraulic efficiency and safety in root systems. In our case, vessel diameter and root hydraulic conductivity were positively related, similar to what was found for stem traits [14], where trees with wider diameters have greater hydraulic conductivity potential and more efficient transport. However, species with larger vessels (*Alchornea glandulosa*, *Mollia gracilis*, *Pourouma cecropiifolia*, *Ocotea* sp.) face increased vulnerability to cavitation under drought stress [14,15,43].

The negative correlation between root vessel diameter and wood specific density aligns with global patterns documented across tropical forests [44,45]. Species combining small root vessel diameters with high wood density (*Leonia crassa*, *Aspidosperma rigidum*, *Warszewiczia coccinea*) achieve greater hydraulic safety and resistance to physical damage, representing a coordinated defensive strategy [14,46]. Similar results were documented for stem traits in 144 plant species from three forests in Colombia [47], in which they state that the larger the pore diameter, the lower the pore density, as a strategy to ensure hydraulic safety.

The overall pattern of principal component analysis (PCA) between above- and below-ground functional traits indicates a diverse set of strategies employed by the studied species. The order in which the species are grouped (Figure 3B) follows a gradient from species with acquisitive strategies, through species with intermediate strategies, to species with conservative strategies, where variation in functional traits such as increased wood density and increased number of vessels per square millimeter are observed. In addition, the differences between the analyzed functional traits among tropical tree roots suggest a variation in resource acquisition and allocation strategies (nutrients, light) with a trade-off between investment in rapid growth for early successional species and investment in higher wood density for late successional species [24,48].

Finally, it is important to mention that, although our study allows us to understand the behavior of the anatomical traits of tropical forest species’ roots, the variation in these traits can also be influenced by environmental variables such as temperature, precipitation, altitude [36,48,49] and soil properties [25,48,50]. In addition, mycorrhizal fungal associations have been documented to influence root morphological traits in different tropical regions [51,52], which could also be extrapolated to the anatomical features of roots.

Future research should prioritize controlled gradient experiments to disentangle genetic from environmental sources of trait variation. Specifically, we recommend: (1) common garden experiments with species representing different hydraulic strategies to isolate genetic components; (2) reciprocal transplant studies across environmental gradients to quantify plasticity; (3) investigation of mycorrhizal associations and their influence on root anatomical traits; and (4) long-term monitoring of hydraulic performance under varying precipitation regimes to validate the adaptive significance of different strategies [53,54].

## 4. Materials and Methods

### 4.1. Study Area

**Figure 4:**
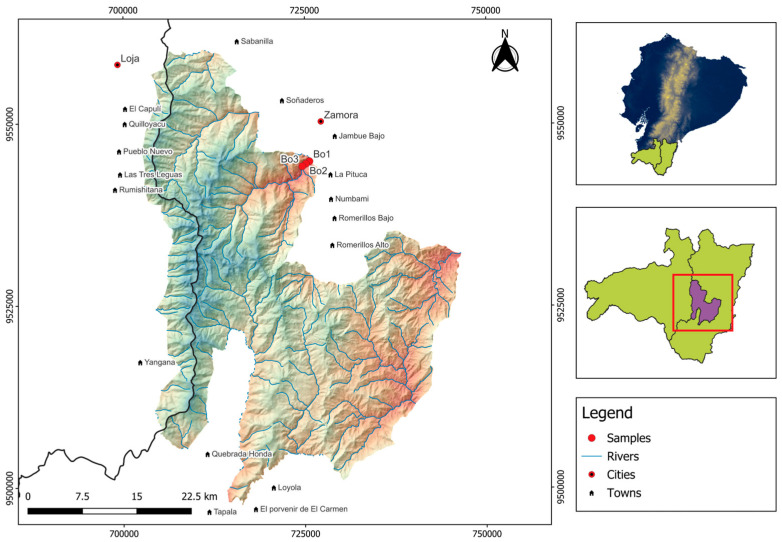
Location of the study area within the Podocarpus National Park, Bombuscaro sector, southern Ecuador. Map shows the three 1-ha permanent plots (marked with red points) where root sampling was conducted.

The study was conducted in a premontane tropical forest of the Podocarpus National Park (4°7′ S, 78°58′ W) located in the Bombuscaro sector between 1030 and 1210 m above sea level (asl) (Figure 4).

The site receives 2000 mm annual precipitation with no clear seasonality and potential evapotranspiration of ~1100 mm/year, maintaining a positive water balance year-round [33]. Mean annual temperature is 19.4 °C with relative humidity consistently above 85% [34]. Soils are eutric or dystric cambisols derived from weathered Jurassic granodiorite or Paleozoic metamorphic schists and sandstones [35].

The forest was classified as evergreen premontane rainforest and harbors high tree diversity with >250 tree species (DBH ≥ 10 cm), most common families (Moraceae, Euphorbiaceae, Lauraceae, Rubiaceae) account for ~35% of stems >10 cm DBH [55]. Our study species represent this taxonomic and functional diversity (Appendix B). Canopy height ranges from 20 to 25 m, with emergent trees reaching 40 m. Root samples were collected during March 2018–November 2019, and laboratory analysis was carried out between January and August 2020.

### 4.2. Design and Data Collection

A forest inventory was carried out in three permanent plots of 1 ha each in Bombuscaro, selecting portions of mature forest with no visible signs of human disturbance. We selected 20 tree species based on: (1) relative abundance of total stems >10 cm DBH within the plots; (2) representation of major plant families; and (3) coverage of contrasting life history strategies ranging from fast-growing pioneers (e.g., *Alchornea glandulosa*, *Mollia gracilis*, *Pourouma cecropiifolia*) to late successional species (e.g., *Aspidosperma rigidum*, *Leonia crassa*), as well as understory specialists to emergent canopy trees (Appendix B). These 20 species represented 41% of the total basal area and 38% of the stems in the study plots.

From each species, 3 to 8 adult individuals (DBH > 10 cm), were selected using a stratified random sampling approach within each 1-ha plot. Root samples were taken by manually excavating up to 20 cm deep into the soil to reach the main lateral roots. We targeted roots with a diameter of 4 to 8 mm for anatomical analysis, following McCormack et al. [6] classification of “transport fine roots” (2–5 mm diameter) and “small woody roots” (5–10 mm diameter), which are functionally distinct from absorptive fine roots (<2 mm) in their anatomical structure and hydraulic properties. Thick roots were carefully traced from the stem base to increasingly thinner lateral roots until reaching the target diameter range (4–8 mm) to ensure species identity and avoid sampling root fragments of uncertain origin. Tree root segments of 3–5 cm length were stored in 70% ethanol at 4 °C in labeled glass vials sent to Göttingen for anatomical analysis. The sampling procedure was designed to minimize ecological disturbance, excavations were immediately backfilled with original soil.

We analyzed four root anatomical traits across 20 tree species. Sample sizes ranged from 3 to 8 individuals per species (mean = 7 individuals per species; see Appendix A for species-specific sample sizes). The analyzed traits were: vessel diameter (Vdia), vessel density (Vdens), vessel lumen fraction (Vlumen fraction), and hydraulic conductivity (Ks), which control water and nutrient transport capacity in woody roots [30,31,56].

**Figure 5:**
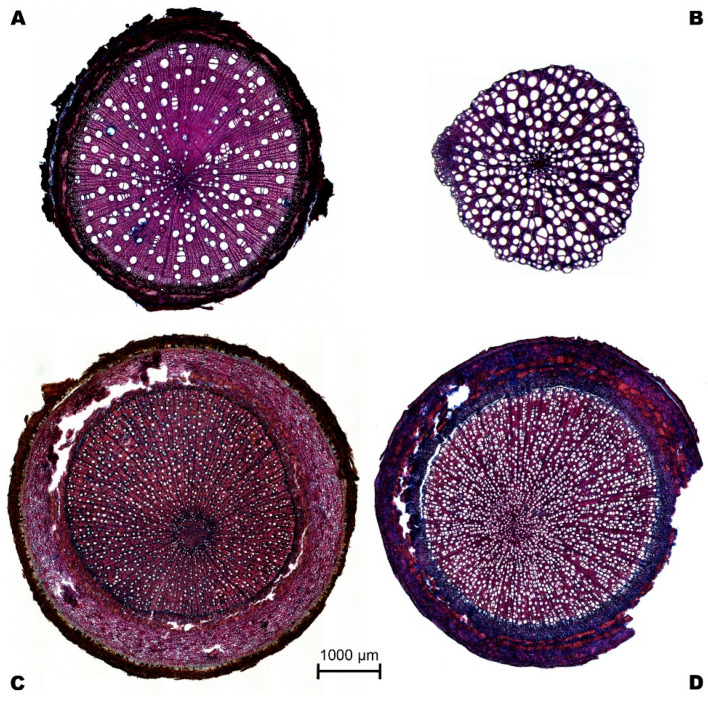
Example of cross-sections of roots of four tree species: (**A**). *Alchornea glandulosa*, (**B**). *Mollia gracilis*, (**C**). *Aspidosperma rigidum*, and (**D**). *Leonia crassa*.

To determine the anatomical dimensions of the collected roots, transverse sections from 5 to 20 μm thick were made using a sliding microtome (G.S.L.1, WSL, Birmensdorf, Switzerland). Once the cross-sections were obtained, the samples were stained with chemical solutions (safranin and astrablue) [31]. Visualization of the anatomical elements of the roots was performed using a stereomicroscope with a digital camera (Zeiss SteREOV20, Carl Zeiss MicroImaging GmbH, Göttingen, Germany) (Figure 5). The images were processed with Adobe Photoshop CS6 (version 13.0.1, Adobe Systems Incorporated, USA) and the software ImageJ (version 1.47, https://imagej.net/ij/) using the particle analysis function to estimate average vessel diameter and vessel density.

In addition to the anatomical traits of the roots, one trait associated with the stem was sampled: wood specific gravity (WSG). To determine WSG a cylindrical wood core was collected from each individual at breast height (1.30 m above ground level). Each core measured approximately 5 cm in length and 5.15 mm in diameter and was extracted using an increment borer (Haglöf, Langsele, Sweden) inserted perpendicular to the trunk surface. WSG was calculated by dividing the dry weight of each core by its green volume (g/cm^3^).

Finally, we used the following leaf characteristics available for the same tree individuals sampled in this study from Homeier et al. [57]: Specific leaf area (SLA, cm^2^ g^−1^) was calculated by dividing total leaf area of 20 leaves by leaf dry mass of these leaves. Leaf toughness (kN m^−1^) was estimated as the mean of six punch tests using a digital penetrometer (fat-ended 2.0 mm diameter steel punch, DS-50 N, Imada Inc., Toyohashi, Japan) on three fresh leaves (excluding the midrib and other major veins). Leaf nitrogen (N) was determined from the leaves collected for SLA [25,57].

Hydraulic conductivity was calculated using the Hagen-Poiseuille equation:

$$Ks=(\pi \times \rho \times \sum_{i=1}^{n}{d_{i}}^{4})/(128\eta \times A_{Xylem})$$

where ρ is the density of water, η is the viscosity of water, d_i_ is vessel diameter of each single vessel *i*, and n is the number of vessels per unit cross-sectional xylem area (*A_Xylem_*) [14,30,31,58].

### 4.3. Data Analysis

To visualize interspecific variation in root anatomical traits (vessel diameter, vessel density, vessel lumen fraction, and hydraulic conductivity) among the 20 trees species, box plots were constructed showing median values. We performed one-way ANOVA followed by Tukey’s HSD post hoc tests to compare traits among ecological groups (acquisitive, intermediate, conservative) We also analyzed correlations between roots anatomical traits and aboveground functional traits (WSG, SLA, Leaf N) by means of a correlation matrix, using Pearson’s correlation coefficient to determine significant differences, after confirming with the Shapiro–Wilk test (*p*-value < 0.05) that the data came from a standard distribution.

Finally, two Principal Component Analyses (PCA) were carried out: the first one to visualize how the species are grouped according to the anatomical features of the roots, and the second one to visualize how the species are grouped according to the features of the roots, stem, and leaves. All analyses were performed using the programming language R version 3.5.1. [59].

## 5. Conclusions

This study demonstrates that root anatomical traits constitute an independent axis of functional variation from leaf economic traits, challenging the assumption of coordinated whole-plant strategies in the studied tropical premontane tree species. The predominance of intermediate hydraulic strategies among most species (60%) suggests that ecological generalism may be more adaptive than extreme specialization in diverse tropical forest ecosystems.

Three key findings emerge from our analysis: First, the orthogonality between above- and belowground trait dimensions indicate that plants can optimize leaf and root functions through decoupled evolutionary pathways. Second, the vessel diameter-density trade-off represents a fundamental constraint on root hydraulic architecture, with species balancing efficiency versus safety according to their ecological requirements. Third, the strong coordination between root anatomical traits and wood density reveals integrated structural strategies that span the plant’s hydraulic continuum from roots to stems.

These findings advance our understanding of plant functional ecology by demonstrating that belowground traits operate as independent drivers of species coexistence and ecosystem functioning in tropical forests. The prevalence of compensatory strategies over extreme specialization suggests that maintaining functional flexibility may be crucial for species persistence under environmental variability. This research provides a foundation for predicting how tropical tree communities may respond to changing environmental conditions, particularly those affecting water availability and resource competition.

## Data Availability

Data is contained within the article.

## Appendix A. Average Values of the Properties Studied in 20 Tree Species

**Table A1:** Studied tree species with number of replicates and average root properties.

Species	N° Ind.	Alumen (%)	Vdens (n mm^−2^)	Vdia (µm)	Ks (kg m^−1^ MPa^−1^ s^−1^)	WSG (g cm^−3^)
*Saurauia* sp.	8	14.06	55.77	52.23	18.64	0.36
*Guatteria pastazae*	6	11.46	32.08	57.70	39.10	0.47
*Aspidosperma rigidum*	8	18.42	194.92	34.32	12.09	0.68
*Chrysochlamys membranacea*	8	15.78	53.75	57.00	28.30	0.43
*Alchornea glandulosa*	8	24.20	89.05	61.32	73.87	0.39
*Pseudopiptadenia suaveolens*	8	8.91	27.81	59.18	25.01	0.60
*Ocotea* sp.	8	14.15	41.73	67.55	34.10	0.41
*Grias peruviana*	5	14.40	75.06	48.01	20.23	0.62
*Mollia gracilis*	8	14.25	42.51	61.25	54.58	0.50
*Miconia aff punctata*	8	11.61	105.91	37.23	11.08	0.56
*Guarea macrophylla*	8	12.10	78.45	39.72	17.77	0.51
*Clarisia racemosa*	8	14.93	61.47	53.62	30.63	0.59
*Pseudolmedia laevis*	6	13.71	55.55	50.96	21.17	0.70
*Otoba parvifolia*	7	12.77	49.70	53.75	17.53	0.40
*Hieronyma oblonga*	5	17.95	86.75	48.23	23.02	0.56
*Ladenbergia oblongifolia*	7	15.62	93.44	45.92	20.21	0.49
*Warszewiczia coccinea*	8	9.50	105.01	31.45	5.51	0.54
*Pouteria torta*	7	10.09	60.22	43.00	13.29	0.79
*Pourouma cecropiifolia*	3	13.24	33.82	63.44	35.50	0.37
*Leonia crassa*	7	22.42	185.49	37.93	14.87	0.52

## Appendix B. Listing of Species According to Their Ecological Strategies

**Table A2:** Studied tree species and their ecological strategies.

Family	Species	Succession Status: Early (E), Intermediate (I), Late (L).	Life Strategy: Acquisitive (A), Intermediate (I), Conservative (C).	Size: Undergrowth (U), Intermediate (I), Canopy (C).
Actinidiaceae	*Saurauia* sp.	E	A	U
Annonaceae	*Guatteria pastazae*	I	I	C
Apocynaceae	*Aspidosperma rigidum*	L	C	C
Clusiaceae	*Chrysochlamys membranacea*	L	C	U
Euphorbiaceae	*Alchornea glandulosa*	E	A	I
Fabaceae	*Pseudopiptadenia suaveolens*	I	I	C
Lauraceae	*Ocotea* sp.	L	C	C
Lecythidaceae	*Grias peruviana*	L	C	U
Malvaceae	*Mollia gracilis*	E	A	C
Melastomataceae	*Miconia aff punctata*	E	I	U
Meliaceae	*Guarea macrophylla*	I	I	U
Moraceae	*Clarisia racemosa*	L	C	C
Moraceae	*Pseudolmedia laevis*	I	I	C
Myristicaceae	*Otoba parvifolia*	E	A	C
Phyllanthaceae	*Hieronyma oblonga*	I	I	C
Rubiaceae	*Ladenbergia oblongifolia*	I	I	C
Rubiaceae	*Warszewiczia coccinea*	L	I	I
Sapotaceae	*Pouteria torta*	L	C	C
Urticaceae	*Pourouma cecropiifolia*	E	A	I
Violaceae	*Leonia crassa*	L	C	C

## Appendix C. Principal Component Analysis Loading Scores

**Table A3:** Variable loadings for PCA based on root anatomical traits (Figure 3A).

Variable	PC1	PC2
Vessel diameter	+0.89	−0.12
Vessel density	−0.76	+0.82
Vessel lumen fraction	+0.45	+0.76
Hydraulic conductivity	+0.91	−0.08

**Table A4:** Variable loadings for PCA including aboveground and belowground traits (Figure 3B).

Variable	PC1	PC2
**Vessel diameter**	−0.68	+0.62
**Vessel density**	+0.71	−0.23
**Vessel lumen fraction**	+0.58	+0.34
**Hydraulic conductivity**	−0.73	+0.58
**Stem wood density**	+0.79	−0.18
**Specific leaf area**	−0.15	+0.81
**Leaf nitrogen**	−0.09	+0.77

Note: Loadings >0.70 are considered strong and are shown in bold in the original analysis.
